# Inactivation of Pepper Mild Mottle Virus in Water by Cold Atmospheric Plasma

**DOI:** 10.3389/fmicb.2021.618209

**Published:** 2021-01-28

**Authors:** Arijana Filipić, David Dobnik, Magda Tušek Žnidarič, Bojana Žegura, Alja Štern, Gregor Primc, Miran Mozetič, Maja Ravnikar, Jana Žel, Ion Gutierrez Aguirre

**Affiliations:** ^1^Department of Biotechnology and Systems Biology, National Institute of Biology, Ljubljana, Slovenia; ^2^Jožef Stefan International Postgraduate School, Ljubljana, Slovenia; ^3^Department of Genetic Toxicology and Cancer Biology, National Institute of Biology, Ljubljana, Slovenia; ^4^Department of Surface Engineering, Jožef Stefan Institute, Ljubljana, Slovenia; ^5^University of Nova Gorica, Nova Gorica, Slovenia

**Keywords:** enteric viruses, pepper mild mottle virus, virus inactivation, water decontamination, cold atmospheric plasma

## Abstract

Water scarcity is one of the greatest threats for human survival and quality of life, and this is increasingly contributing to the risk of human, animal and plant infections due to waterborne viruses. Viruses are transmitted through polluted water, where they can survive and cause infections even at low concentrations. Plant viruses from the genus *Tobamovirus* are highly mechanically transmissible, and cause considerable damage to important crops, such as tomato. The release of infective tobamoviruses into environmental waters has been reported, with the consequent risk for arid regions, where these waters are used for irrigation. Virus inactivation in water is thus very important and cold atmospheric plasma (CAP) is emerging in this field as an efficient, safe, and sustainable alternative to classic waterborne virus inactivation methods. In the present study we evaluated CAP-mediated inactivation of pepper mild mottle virus (PMMoV) in water samples. PMMoV is a very resilient water-transmissible tobamovirus that can survive transit through the human digestive tract. The efficiency of PMMoV inactivation was characterized for infectivity and virion integrity, and at the genome level, using test plant infectivity assays, transmission electron microscopy, and molecular methods, respectively. Additionally, the safety of CAP treatment was determined by testing the cytotoxic and genotoxic properties of CAP-treated water on the HepG2 cell line. 5-min treatment with CAP was sufficient to inactivate PMMoV without introducing any cytotoxic or genotoxic effects in the *in-vitro* cell model system. These data on inactivation of such stable waterborne virus, PMMoV, will encourage further examination of CAP as an alternative for treatment of potable and irrigation waters, and even for other water sources, with emphasis on inactivation of various viruses including enteric viruses.

## Introduction

Globalization, urbanization, climate change, and lack of correct wastewater treatment are contributing to a steady decrease in the availability of clean water in many regions of the world (Thebo et al., 2017). Water-transmitted viruses are highly stable and many can survive long times in an aqueous environment, eventually reaching sources of potable water, irrigation water, or seafood culture sites in coastal waters. These can then serve as the infection routes for humans, animals and plants, making waterborne viruses an increasing concern (Mehle et al., 2018b). Metagenomic analysis has revealed the presence of rich viral diversity in wastewater samples, where human enteric viruses and plant viruses from the genus *Tobamovirus* were among the most relevant human and plant viruses, respectively, (Bačnik et al., 2020; Martínez-Puchol et al., 2020).

Tobamoviruses are highly mechanically transmissible rod-shaped viruses of the *Virgaviridae* family that can infect a variety of economically important crop plants, such as tomato, pepper, cucumber, melon, and watermelon, to cause huge losses^1^. Tomato brown rugose virus (ToBRFV) is at present one of the most important members of this genus, and it is an expanding global threat for tomato crops worldwide due to the emergence of resistance breaking strains (Salem et al., 2016; Luria et al., 2017). Another tobamovirus, cucumber green mottle mosaic virus (CGMMV), is also re-emerging and causing significant losses of cucurbit crops (Dombrovsky et al., 2017). Reports of infective tobamoviruses in treated wastewater (Bačnik et al., 2020) or environmental waters (Jeżewska et al., 2018) and their survival in the nutrient solution for up to 6 months (Pares et al., 1992) suggest that water might have an important role in the epidemiology and transmission of these viruses. This has been further confirmed by studies that have reported water-mediated transmission of CGMMV (Li et al., 2016) and other tobamoviruses, and this becomes especially relevant for crops grown using hydroponics (Mehle and Ravnikar, 2012).

Pepper mild mottle virus (PMMoV) is a tobamovirus very closely related to the above-mentioned ToBRFV and CGMMV. PMMoV causes disease in pepper plants, and as for other tobamoviruses, it has recently been confirmed to remain infective after wastewater treatment in a WWTP (Bačnik et al., 2020). This represents a risk for regions where reclaimed water is used for irrigation (Thebo et al., 2017). Furthermore, PMMoV is such a resilient virus that it can survive the harsh environment of the human gastrointestinal tract, and it remains infectious even after excretion in feces, where it has been shown to be the most dominant virus, depending on nutritional habits (Zhang et al., 2006). This is why PMMoV has emerged as a link between waterborne tobamoviruses and enteric viruses. PMMoV is present worldwide in water matrices that come into contact with human fecal pollutants, from treated wastewaters to rivers and seawater, where it shows minimal resistance to changes in the environment (Kitajima et al., 2018; Symonds et al., 2018; Bivins et al., 2020). Concentration of PMMoV in wastewaters is usually high, ranging from 10^6^ to 10^10^ cp L^–1^, which enables its consistent detection needed for successful monitoring. Additionally, PMMoV presence in wastewater correlates with the presence of various enteric viruses. Since the occurrence of PMMoV in wastewaters is tightly connected to the dietary customs and not to the acute viral infection of the population, it has low seasonal variations (Symonds et al., 2018, 2019). For these reasons, PMMoV has been suggested as an indicator of water fecal pollution (Rosario et al., 2009; Symonds et al., 2016, 2018; Bivins et al., 2020), and it has also been used as an enteric virus surrogate to test the efficiency of water treatment methods (Kitajima et al., 2018; Symonds et al., 2019; Tandukar et al., 2020).

Water-transmissible tobamoviruses pose major threats to plant health, which calls for efficient, clean, and cost-efficient methodologies for their inactivation. Methods for waterborne virus inactivation usually depend on the use for the water that needs to be treated; e.g., for drinking water or pools, chlorination is often the method of choice, while domestic wastewater goes through WWTPs, where different levels of treatment are applied. However, tobamoviruses are often not inactivated by conventional water treatments (Bačnik et al., 2020), and in addition, many of the currently available virus inactivation methods are not exempt from caveats, such as the generation of toxic intermediates, the demand for large infrastructure and frequent maintenance, or the high cost (Stewart-Wade, 2011). One method that has recently shown great potential for water decontamination is cold atmospheric plasma (CAP; Filipić et al., 2020).

Plasma is the fourth and most abundant state of matter in the visible universe. It is partially or completely ionized gas that is abundant with charged particles, molecules and atoms in their ground or excited states, UV photons and reactive species (Filipić et al., 2020). Plasma can be either thermal, where all of the particles have approximately the same temperature, or cold, where electrons have much higher temperatures than heavier ions, neutral atoms, or molecules. Cold plasma can be further divided into low-pressure and atmospheric pressure (Mozetič et al., 2019). CAP can be sustained either in a continuous mode (using radiofrequency or microwave discharges of frequencies from about 1 MHz to several GHz), or in the form of stochastically generated streamers of duration of a microsecond using high-frequency discharges in the range from 1 kHz to a few tens of kHz. These latter are often preferred, as the gas is easily kept close to room temperature, and the concentration of the reactive plasma species is high as long as the streamer lasts. In this way, at the point of application, CAP is at room temperature, which makes it useful for treatment of biological samples, as it does not cause thermal damage. Some CAP constituents have strong antimicrobial properties, such as reactive oxygen and nitrogen species (RONS) and UV radiation (Guo et al., 2015). This has motivated its use for decontamination in various fields, including medicine (Sakudo et al., 2019) and food production (Bourke et al., 2018). Most studies to date have targeted bacterial inactivation, while fewer studies have considered virus inactivation (reviewed by Filipić et al., 2020) and of these, only two studies examined virus inactivation in larger volumes (up to few mL) of water (Guo et al., 2018; Filipić et al., 2019).

In the present study, we evaluated the applicability of CAP for inactivation of PMMoV in water samples. PMMoV was used as a representative resilient tobamovirus, i.e., plant virus. Viral inactivation was characterized at different levels, using test plant infectivity assays, transmission electron microscopy (TEM) and PCR-based molecular methods. As CAP produces a myriad of RONS that diffuse into the water during treatment, we examined the suitability of CAP for human-related uses through determining the cytotoxic and genotoxic activities of CAP-treated water using MTS and alkaline comet assays, respectively, with a human hepatocellular carcinoma (HepG2) cell line.

## Materials and Methods

### Preparation of Water Samples With and Without PMMoV

Virus-containing plant homogenates diluted in water (PMMoV samples) were used to study the effects of CAP and control treatments on virus inactivation, while virus-free homogenates diluted in water (PMMoV-free samples) were used for assessment of cytotoxic and genotoxic properties of CAP-treated water.

Pepper mild mottle virus samples were prepared from frozen leaf tissue infected with PMMoV from a collection at the National Institute of Biology (isolate no. 272). The tissue was homogenized in inoculation buffer (20 mM phosphate buffer, 2% polyvinylpyrrolidone [molecular mass 10,000 Da]) in a mass to volume ratio of approximately 1:10, using extraction bags (Bioreba, Switzerland). The resulting homogenate was gently applied to three leaves of pepper plants that had been prior dusted with carborundum powder, to generate microinjuries to the leaves. After 7 min, the inoculum was rinsed off with tap water, and the plants were grown for 21 days. Symptomatic leaf tissue without necrotic regions was collected and cut into small pieces of a few square millimeters, which were then thoroughly mixed, aliquoted into microcentrifuges, and stored at −20°C. The presence of PMMoV in these aliquots was confirmed by RT-qPCR (see section “RT-qPCR”). PMMoV samples were prepared by homogenization of 50 ± 5 mg of aliquoted frozen leaf tissue in 1 mL tap water, using a FastPrep system (MP Biomedical, France), and then further diluted 1,000 times. Ten milliliters of the prepared PMMoV samples were used for the CAP and control treatments. One PMMoV sample was always left untreated, which served as a PMMoV-positive control (PMMoV-PC) for infectivity assays, TEM, RT-PCR, and RT-droplet digital PCR (RT-ddPCR).

For cytotoxicity and genotoxicity assays, PMMoV-free samples were prepared as described above, with the exception that the sampled plant was a healthy, non-inoculated pepper plant. Ten milliliters of PMMoV-free samples were treated using CAP, and the potential cytotoxic and genotoxic properties of this CAP-treated water were examined. The PMMoV-free sample that remained untreated was used as the PMMoV-free negative control (PMMoV-free-NC).

### CAP and Control Treatments

**FIGURE 1:**
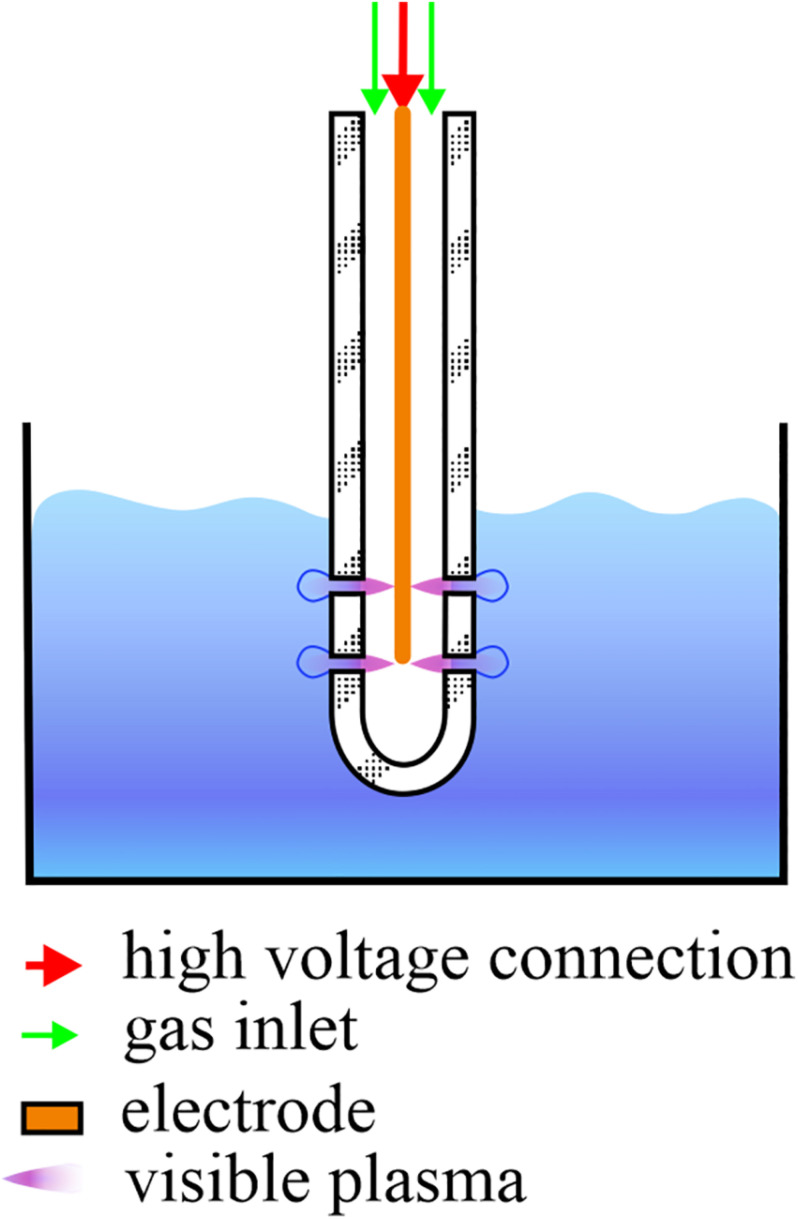
Schematic representation of a single-electrode cold atmospheric plasma (CAP) jet. During CAP treatment of the infected water samples, the electrode and glass tube are submerged in 10 mL samples and CAP streamers can be seen. Streamers enter the samples in the form of bubbles through the four openings, two on each side of the glass tube.

For the CAP treatments, an atmospheric pressure plasma jet in single electrode configuration was used (Filipić et al., 2019). The electrode was placed in a glass tube and submerged in the PMMoV sample or PMMoV-free sample. CAP was introduced into the samples in the form of bubbles created by the gas flow that left the tube through the four openings at the end of the tube (Figure 1). CAP was ignited in a mixture of 99% argon and 1% oxygen, with constant gas flow of 1.7 ± 0.1 L min^–1^. The electrode was connected to a frequency generator of 31 kHz that operated at a peak-to-peak voltage of 6 kV, with total average output power of ∼3 W. For assessment of both PMMoV inactivation by CAP and the toxic effects of CAP-treated water, based on preliminary trials the treatment duration was 5 min or 3 min, which was applied as three independent repetitions.

To confirm that virus inactivation was the result of CAP treatment, four control treatments were performed using 10 mL PMMoV samples. The first control treatment included only stirring on the magnetic stirrer for 5 min. The second control treatment was completed after 5 min by flowing the same gas mixture used for CAP generation (99% argon, 1% oxygen) into the PMMoV sample, but in the absence of the electrical discharge. The other two control treatments consisted of addition of hydrogen peroxide (H_2_O_2_) at two concentrations, which reflected those found with CAP treatments (Table 1), as 2.5 mg L^–1^ and 5 mg L^–1^ for 5 min with constant stirring.

**TABLE 1:** Semi-quantitative H_2_O_2_ measurements following the cold atmospheric plasma treatments.

Treatment time (min)	H_2_O_2_ concentration (mg L^–1^)
3	2–5
3	∼2
3^*a*^	∼2
5	∼5
5	∼5
5	∼5

*^*a*^Only partial inactivation achieved.*

The CAP-treated and control-treated PMMoV samples and PMMoV-PC were used directly in test plant infectivity assays and for preparing grids for TEM. In addition, aliquots of CAP-treated or control-treated PMMoV samples and PMMoV-PC were frozen in liquid nitrogen and stored at −80°C until RNA extraction was carried out, using RNeasy plant mini kit (Qiagen, Germany), according to the manufacturer‘s instructions, although without using mercaptoethanol. Luciferase RNA (2 ng per sample) was added to the samples prior to the extraction as the external control of extraction. A negative control of extraction that consisted of RNAse-free water was included among the extracted samples, to monitor for possible contamination.

### Test Plant Infectivity Assays

To determine the efficiency of virus inactivation, test plant infectivity assays were performed using pepper plants (*Capsicum annum*). Two leaves of six to eight plants were mechanically inoculated (see section “Preparation of water samples with and without PMMoV”) with CAP-treated or control-treated PMMoV samples or PMMoV-PC, at least 4 weeks after sowing. A negative control for the procedure consisted of plant inoculation with 5 min CAP-treated tap water without any virus. Each experiment also included plants that were not inoculated at all, which served as controls for potential contamination during the routine watering at the greenhouse. All of the plants were grown in a quarantine greenhouse at 22 ± 2°C during the light period (16 h), and 19 ± 2°C during the dark period (8 h). The test plants were examined regularly for development of symptoms of viral infection and were sampled at least 2 weeks post inoculation. The plant material collected from plants inoculated with the same sample and the plant material from non-inoculated plants were pooled together and the RNA was extracted with RNeasy plant mini kit (see section “CAP and control treatments”). After RNA extraction, the presence/absence of PMMoV in the analyzed samples was confirmed using RT-qPCR (section “RT-qPCR”). Viruses were considered infective when they were detected with RT-qPCR in the upper, non-inoculated leaves. For the pools of plants inoculated with CAP-treated PMMoV samples that gave a positive signal by RT-qPCR, each individual plant was tested again for the virus either with ImmunoStrip for PMMoV (Agdia, IN, United States) or by RT-qPCR.

### Transmission Electron Microscopy

The presence of virus particles and changes in their morphology after the CAP and control treatments were examined using TEM. To observe the effects of very short CAP treatments on PMMoV virions, an additional CAP treatment of 1 min was included in the TEM measurements. CAP-treated and control-treated PMMoV samples and PMMoV-PC were applied to freshly glow-discharged grids. After 5 min, the samples were soaked away and stained with 1% (w/v) water solution of uranyl acetate. The grids were examined by TEM (Philips CM100; FEI) that was operated at 80 kV, and micrographs were captured using a CCD camera (Orius SC 200; Gatan Inc., CA, United States), with the Digital Micrograph software (Gatan Inc., United States).

### Molecular Methods

#### RT-qPCR

RT-qPCR was performed to confirm the presence/absence of the virus and its systemic spread in the inoculated test plants. The extracted RNA was analyzed with RT-qPCR using two assays: one for PMMoV detection (Rački et al., 2014), and the other for detection of luciferase, an external control for the RNA extraction (Toplak et al., 2004). AgPath-ID One-Step RT-PCR kit was used (Life Technologies, CA, United States). The final reaction volume was 10 μL, which included 2 μL sample (e.g., extracted RNA), primers and probe, and the rest of the components as recommended by the manufacturer. The PMMoV assay included primers (Haramoto et al., 2013) and probe (Zhang et al., 2006) at 900 and 200 nM, respectively, while the luciferase assay included 1,000 nM primers and 500 nM probe (Toplak et al., 2004). To test the success of the amplification and for potential contamination during RT-qPCR preparation, a positive control of RT-qPCR (previously extracted and characterized PMMoV RNA) and a non-template negative control (sterilized water) were used, respectively. The reactions were run in duplicate, and all of the RNA samples were analyzed undiluted and at 10-fold dilution, to allow for possible inhibitory effects. The cycling conditions were: 10 min at 48°C, 10 min at 95°C, and 45 cycles of 15 s at 95°C and 1 min at 60°C. The reactions were run on an ABI Prism 7900 HT Fast Detection system (Applied Biosystems, MA, United States) or a QuantStudio 7 Flex Real-Time system (Applied Biosystems), and the results were processed using the SDS 2.4 software (Applied Biosystems) or the QuantStudio Real-Time PCR software v 1.3 (Applied Biosystems), respectively.

#### RT-ddPCR

RT-droplet digital PCR was used to determine the concentrations of the viral RNA in the PMMoV-PC and treated PMMoV samples, with the same PMMoV specific set of primers and the probe as described above. One-Step RT-ddPCR advanced kit for probes (Bio-Rad, CA, United States) was used, and the reactions were run in duplicate. The final reaction volume of 20 μL consisted of 4 μL sample (e.g., extracted RNA), 900 nM primers, and 250 nM probe, with the rest of the components as recommended by Bio-Rad. The cycling conditions were as follows: 60 min at 50°C, 10 min at 95°C, 40 cycles of 30 s at 95°C and 1 min at 56°C, and 10 min at 98°C. Sterilized water was used as a non-template control for the RT-PCR reactions, to monitor for possible contaminations of the RT-ddPCR reagents. For the generation of droplets, an automated droplet generator (Bio-Rad) was used. The droplets were read using a droplet reader (QX100 or QX200; Bio-Rad), and the data were processed using the QuantaSoft software, version 1.7.4 (Bio-Rad). The RT-ddPCR was processed as described by Mehle et al. (2018a) with the modification that the thresholds were set manually.

#### RT-PCR

To examine the effects of CAP and the control treatments on the integrity of the viral RNA, three long fragments that together almost completely covered the viral genome were amplified by RT-PCR. The three sets of primers were designed in-house using Primer-BLAST (NCBI, United States; Table 2). One-Step RT-PCR kit was used (Qiagen, Germany) without the Q-solution, according to the manufacturer instructions, with minor modifications; namely, smaller reaction volumes (25 μL) were prepared, which included 5 μL template RNA. The cycling conditions were: 30 min at 50°C, 15 min at 95°C, 35 cycles of 30 s at 94°C, 60 s at 52°C and 95 s at 72°C, 10 min at 72°C, and an infinite hold at 4°C. Sterilized water was used as the non-template control for the RT-PCR reactions, to monitor for possible contaminations of the reagents. The amplified products were detected using agarose gel electrophoresis, with 1% agarose gel run for 45 min at 100 V in TAE buffer. Ethidium bromide was used for visualization of the amplified fragments, and fragment sizes were estimated using a 1-kb ladder.

**TABLE 2:** Primers used in the RT-PCR.

Primer set	Length of amplified product (bp)	Position in genome	Primer sequence
#1	1,465	159–178	FW: 5′-ACTGTACGAATCAG CGGTCG-3′
		1,623–1,601	R: 5′-TTCAAGAGCCTTTTCCG AAACAG-3′
#2	1,580	1,874–1,900	FW: 5′-ATGAGAGTGGTTTGACCT TAACGTTTG-3′
		3,453–3,424	R: 5′-ACCTTTGTACACCGATTCTA TCTGTAATTG-3′
#3	1,614	3,861–3,880	FW: 5′-GCTTTCAAGGGC GAGTTTGG-3′
		5,474–5,456	R: 5′-AGTTCAACGGGTC CTCCTT-3′

*FW, forward oligonucleotides; and R, reverse oligonucleotides.*

*All primers purchased from Integrated DNA Technologies, United States.*

### Temperature, pH and H_2_O_2_ Production

Three physicochemical characteristics of the PMMoV samples were determined before and after each treatment: temperature, pH, and H_2_O_2_ production. The pH measurements were done using test strips (Macherey-Nagel, Germany) and the H_2_O_2_ measurements using semi-quantitative Quantofix Peroxid 25 test strips (Macherey-Nagel, Germany). Temperature was measured with a standard alcohol thermometer.

### Cytotoxic and Genotoxic Effects of CAP-Treated PMMoV-Free Samples

Cytotoxic and genotoxic effects of the CAP-treated PMMoV-free samples were determined using the MTS and alkaline comet assays, respectively. Samples (10 mL) were treated with CAP for 5 min and 3 min, and the assays were conducted immediately after. To ensure aseptic conditions for the *in-vitro* experiments, all of the samples were filtered using commercial 0.2-μm pore filters, directly after the CAP treatments and prior to the cell exposure.

The human hepatocellular carcinoma cell line (HepG2 cells) was obtained from American Type Culture Collection (VA, United States), and served as the model system for cytotoxicity and genotoxicity assessments of CAP-treated PMMoV-free samples. The HepG2 cells were cultured at 37°C and 5% CO_2_ in Eagle’s minimal essential medium with 10% fetal bovine serum, 1% non-essential amino acids, 2 mM L-glutamine, 1 mM sodium pyruvate, 2.2 *g* L^–1^ NaHCO_3_, and 100 IU mL^–1^/10 mg mL^–1^ penicillin/streptomycin. For the MTS and alkaline comet assays, HepG2 cells were seeded at specific densities on assay-specific culture plates and left to attach overnight. The next day, the HepG2 cells were exposed to 5-min and 3-min CAP-treated PMMoV-free samples diluted in growth medium (1:2), for 2 h and 24 h. Other samples were also included in the experiments: PMMoV-free-NC in growth medium (1:2); a negative control (phosphate-buffered saline in growth medium; 1:2); and assay-specific positive controls.

#### Cell Viability Test – The MTS Assay

Changes in the HepG2 cell viability after exposure to CAP-treated PMMoV-free samples were evaluated using the MTS assay, as described by Hercog et al. (2019). The HepG2 cells were seeded at a density of 8,000 cells well^–1^ (40,000 cells mL^–1^) in 96-well plates (Nunc, Naperville IL, United States). After their exposure to the above-mentioned samples, MTS:phenazine methosulfate solution (20:1) was added to each well, and incubated for 3 h. The amount of formazan produced was measured using a microplate reader (Synergy MX; BioTek, United States) at 490 nm. Viability of the exposed cells was calculated as proportions (%) of the PMMoV-free-NC. H_2_O_2_ (100 μg mL^–1^) and etoposide (50 μg mL^–1^) were used as positive controls, for 2 h and 24 h, respectively. The GraphPad Prism 8 program (GraphPad Software, United States) was used for data visualization and statistical evaluation. One-way analysis of variance (ANOVA) and Dunnett’s multiple comparison tests were applied to determine statistically significant differences in cell viabilities between tested cell populations. Three independent experiments were performed, each with five replicas.

#### Induction of DNA Strand Breaks – The Alkaline Comet Assay

Induction of DNA strand breaks was analyzed using the alkaline comet assay, as described by Žegura and Filipič (2004). Briefly, the cells were seeded in 12-well plates (Corning, Corning Costar Corporation, NY, United States) at a density of 80,000 cells well^–1^ and exposed to the above-mentioned samples. After 2 h and 24 h exposure, the cells were harvested using trypsinization, and embedded in agarose gel (1%; low melting point agarose) positioned on fully frosted, degreased (i.e., overnight in methanol), and pre-coated (1%; normal melting point agarose) glass slides. The cells were lysed in lysis buffer (100 mM EDTA, 2.5 M NaCl, 10 mM Tris, pH 10, 1% Triton X-100) and left for 1 h at 4°C in the dark. This was followed by 20 min of alkaline unwinding (300 mM NaOH, 1 mM EDTA; pH > 13) at 4°C in the dark, after which the nuclei were exposed to the electric current at 25 V (0.5–1.0 V cm^–1^; pH > 13) for 20 min at 4°C in the dark. The slides were neutralized (400 mM Tris buffer, pH 7.5) for 15 min at 4°C in the dark, and stored at 4°C in a humid container until analysis. The nuclei were stained with GelRed for the image analysis, according to the manufacturer protocol. Images were acquired and analyzed with the Comet IV software (Perceptive Instruments Ltd., Haverhill, United Kingdom), using a fluorescence microscope (Eclipse 800; Nikon, Tokyo, Japan). Etoposide (10, 5 μg mL^–1^, for 2, 24 h, respectively) was used as the positive control. Fifty nuclei were analyzed per experimental point, with experiments independently repeated three times. GraphPad Prism (Kruskal–Wallis nonparametric tests and Dunn’s multiple comparison tests) was used to evaluate statistically significant differences in the proportions (%) of tail DNA between the PMMoV-free-NC and the CAP-treated PMMoV-free sample populations.

## Results

### Virus Inactivation – Test Plant Infectivity Assays

Three independent repetitions of the 5-min and 3-min CAP treatments of the PMMoV samples were carried out. RT-ddPCR was used for estimation of the virus concentrations in PMMoV samples, which was similar across all samples, and ranged from 4.90 × 10^5^ to 5.29 × 10^5^ cp μL^–1^ sample (Table 3). The decay in the infectivity was then estimated by assessing the infection of the pepper plants by the CAP-treated viruses in comparison to the PMMoV-PC samples (Table 3). The viruses were completely inactivated (i.e., they did not infect any of the inoculated plants) after all three independent 5-min treatments and in two of the three 3-min treatments. In the third independent 3-min treatment, there was only partial inactivation, with PMMoV only detected in two out of seven inoculated plants (Table 3). All of the pools of plants inoculated with PMMoV-PC tested positive for PMMoV, while the plants inoculated with CAP-treated tap water without virus or non-inoculated plants were negative as determined by RT-qPCR. Control treatments with stirring, gas only, or H_2_O_2_ at both concentrations had no detrimental effects on the virus infectivity (Table 3).

**TABLE 3:** The influence of different treatments on virus infectivity and RNA degradation.

Treatment	Treatment condition	Initial viral RNA concentration (copies μL^–1^ sample)^*a*^	Viral RNA concentration after treatment (copies μL^–1^ sample)^*b*^	Viral RNA degradation (RT-PCR)^*c*^	Viral infectivity^*d*^ (infected plants/inoculated plants)
Stirring	5 min	5.05 × 10^5^	5.71 × 10^5^	–	8/8
Gas	5 min	5.05 × 10^5^	5.07 × 10^5^	–	8/8
H_2_O_2_	2.5 mg L^–1^; 5 min	5.05 × 10^5^	4.91 × 10^5^	–	8/8
	5.0 mg L^–1^; 5 min	5.05 × 10^5^	5.12 × 10^5^	–	8/8
CAP	3 min	4.90 × 10^5^	1.26 × 10^5^	+	0/8
		5.29 × 10^5^	5.92 × 10^4^	+	2/7
		5.29 × 10^5^	3.96 × 10^4^	+	0/7
	5 min	4.90 × 10^5^	1.08 × 10^5^	+	0/8
		5.29 × 10^5^	2.02 × 10^4^	+	0/7
		5.29 × 10^5^	1.36 × 10^4^	+	0/7

*CAP, cold atmospheric plasma treatment.*

*^*a*^Viral concentration determined for PMMoV-PC; i.e., untreated PMMoV samples (mean of two replicates).*

*^*b*^Viral RNA concentration as mean of two replicates, except 2.5 mg L^–1^ H_2_O_2_ (one replicate used).*

*^*c*^RNA was degraded (+) if there was a noticeable reduction in intensity of the band of ≥1 of the three genome regions amplified by RT-PCR.*

*^*d*^Viruses were infective if viral progeny was detected in at least one plant by RT-qPCR in upper, non-inoculated leaves ≥2 weeks post-inoculation.*

### CAP Effect on Virus Integrity – Transmission Electron Microscopy

**FIGURE 2:**
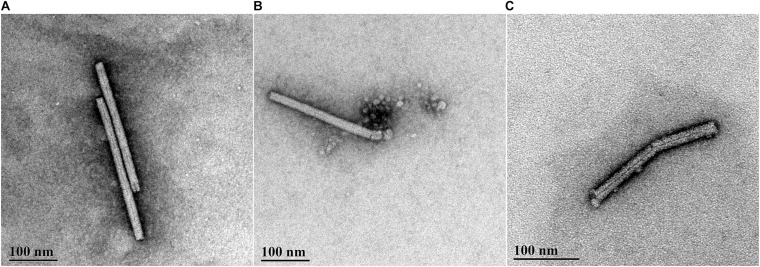
Representative transmission electron microscopy micrographs of pepper mild mottle virus (PMMoV) in water samples. **(A)** Whole, undamaged PMMoV in untreated PMMoV sample (PMMoV-PC). **(B)** Damaged (i.e., broken) PMMoV after 1 min cold atmospheric plasma (CAP) treatment. Structures similar to aggregates are associated with the virus particle, which were not seen for the PMMoV-PC or 5-min and 3-min CAP-treated PMMoV samples; these appear to indicate early viral degradation. **(C)** Damaged PMMoV after 5 min CAP treatment.

The TEM data were in agreement with the data from the infectivity assays. PMMoV-PC contained both whole (see Figure 2A for example) and broken virus particles. The PMMoV samples treated with CAP for 5 min contained lower numbers of viruses than PMMoV-PCs, and the particles were mostly damaged, as shown in Figure 2C. This was also the case for two of the three 3-min treatments. In the third 3-min CAP treatment, where only partial inactivation was achieved, there were more viruses that were not as damaged as in the other CAP-treated PMMoV samples, although they were shorter (230–270 nm) compared to the undamaged PMMoV virions (∼312 nm). In the 1-min CAP-treated PMMoV sample, there were structures similar to aggregates associated with the virus particles (see Figure 2B). These were not observed in the PMMoV-CAP or PMMoV samples treated for 5 min or 3 min, and appear to be indicative of early viral degradation. Control treatments had no effects on the integrity of the virus particles.

### RNA Degradation – Molecular Methods

**FIGURE 3:**
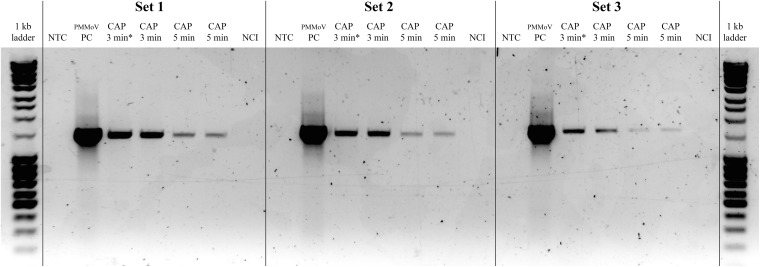
Representative gel showing pepper mild mottle virus (PMMoV) RNA degradation after cold atmospheric plasma (CAP) treatments. Notable degradation compared to PMMoV-PC (positive control; i.e., untreated PMMoV sample) is seen for all three amplified RNA fragments after two independent 5-min and 3-min CAP treatments. NTC, non-template control (i.e., sterilized water); CAP 3 min^∗^, sample with partial PMMoV inactivation; NCI, negative control of isolation (i.e., sterilized water).

The effect of the CAP treatment on the integrity of the viral RNA was assessed by RT-PCR of three long fragments (>1,400 bp), which spanned almost the whole virus genome. RNA degradation was considered positive if the intensity of the agarose gel bands corresponding to at least one of the amplified genomic fragments was notably lower comparing to PMMoV-PC. Viral RNA was degraded in all of the CAP-treated samples, with notable reductions in at least two fragments, as observed for the agarose gel (see Figure 3 for example), while the control treatments had no effects on the viral RNA (Table 3). The viral RNA concentrations measured by RT-ddPCR before and after CAP treatments also showed the decrease by factors of 3.9 to 38.9 (Table 3), with no correlation with the treatment time. This is not surprising as it is known that qPCR and ddPCR are not first line tools to measure genomic degradation as they target very small sequence patches, that can underestimate degradation in other regions of the genome (Rodriìguez et al., 2009).

### Temperature, pH, and H_2_O_2_

No significant changes in pH or temperature were recorded after the CAP or control treatments. After CAP and the gas treatments, a small drop in sample temperature occurred (≤2.5°C), due to the introduction of gas into the samples (i.e., argon and oxygen mixture), and thus enhanced evaporation. H_2_O_2_ concentrations prior to any treatment were always 0 mg L^–1^, and they increased in a time dependent manner to up to ∼5 mg L^–1^ (Table 1).

### Cytotoxic and Genotoxic Activities of CAP-Treated Water

**FIGURE 4:**
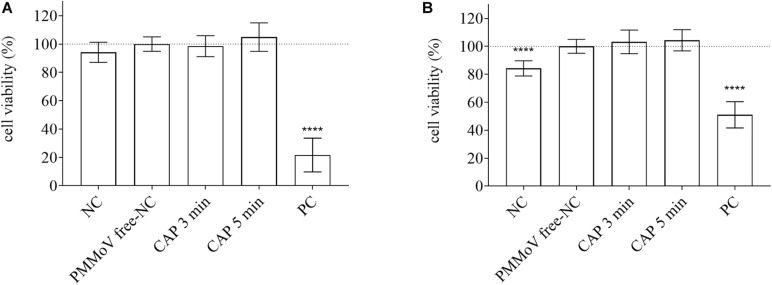
Quantification of viability of HepG2 cells following exposure to cold atmospheric plasma (CAP)-treated pepper mild mottle virus (PMMoV)-free samples. Influence of 5-min and 3-min CAP-treated PMMoV-free samples on viability of HepG2 cells after 2 h **(A)** and 24 h **(B)**, expressed as proportions (%) of PMMoV-free-NC (i.e., untreated PMMoV-free sample) in growth medium (1:2). NC, negative control (phosphate buffered saline: growth medium, 1:2); PC, positive control: H_2_O_2_ (100 μg mL^–1^) for the 2 h time point, and etoposide (50 μg mL^–1^) for the 24 h time point; ****p < 0.0001 (ANOVA and Dunnett’s multiple comparison tests).

**FIGURE 5:**
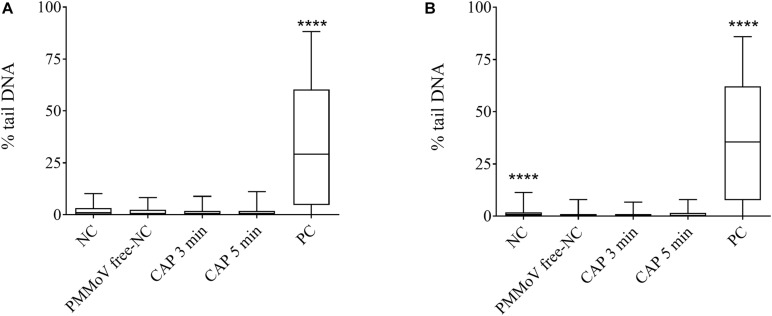
Quantification of genotoxic activity of cold atmospheric plasma (CAP)-treated pepper mild mottle virus (PMMoV)-free samples. Influence of 5-min and 3-min CAP-treated PMMoV-free samples on induction of DNA damage in HepG2 cells after 2 h **(A)** and 24 h **(B)**. Data are expressed as quantile box plots. Edges of the plots represent the 25th and 75th percentiles, with 95% confidence intervals. Solid line through the box is the median. NC, negative control (phosphate buffered saline: growth medium, 1:2); PC, positive control: etoposide (10 μg mL^–1^) for the 2 h time point, and etoposide (5 μg mL^–1^) for 24 h time point. DNA damage induction by CAP-treated PMMoV-free samples was compared to the induction by the PMMoV-free NC (untreated PMMoV-free sample in growth medium [1:2]); ****p < 0.0001 (Kruskal–Wallis nonparametric tests and Dunn’s multiple comparison tests).

For determination of any potential cytotoxic and genotoxic activities of the CAP-treated water, the MTS and alkaline comet assays were performed, respectively. None of the treated samples showed any cytotoxic (Figure 4) or genotoxic (Figure 5) activities against the HepG2 cells. The HepG2 cells exposed to the CAP-treated PMMoV-free samples and the PMMoV-free-NC showed small increases in cell viability and metabolism compared to the negative controls.

## Discussion

Here we report on the use of CAP as a novel, environmentally friendly technology for inactivation of the tobamovirus PMMoV in water. PMMoV is a remarkably stable virus that has been shown to be resistant to various water treatments, including the conventional wastewater treatments used in WWTPs (Kitajima et al., 2018; Bačnik et al., 2020).

While PMMoV causes infections in pepper species (Moury and Verdin, 2012), emerging or re-emerging tobamoviruses closely related to PMMoV, such as CGMMV and ToBRFV, present threats to other economically relevant crops, such as tomato and pumpkin (Dombrovsky et al., 2017; Luria et al., 2017). As irrigation has been described as one of the routes for tobamovirus transmission (Mehle and Ravnikar, 2012; Li et al., 2016; Bačnik et al., 2020), an efficient strategy for inactivation of tobamoviruses in water would help in the design of measures to contain their spread, especially for crops grown using hydroponic systems and in regions where recycled wastewater is used for irrigation (Thebo et al., 2017). In addition, PMMoV has been shown to be similarly, or even more, resistant than enteric viruses when subjected to various water treatment methods (Kitajima et al., 2014; Asami et al., 2016; Schmitz et al., 2016; Lee et al., 2017; Kato et al., 2018; Tandukar et al., 2020). Therefore, it is expected that a water treatment method that can inactivate PMMoV will not only serve for inactivation of tobamoviruses but also for waterborne enteric viruses.

Cold atmospheric plasma has recently emerged as a promising technology for inactivation of microbes, including viruses (reviewed in Filipić et al., 2020). The main focus of studies published to date has been treatment of enteric viruses or their surrogates; i.e., hepatitis A (Bae et al., 2015; Park and Ha, 2018), coxackievirus (Takamatsu et al., 2015), norovirus (Ahlfeld et al., 2015; Aboubakr et al., 2020), adenovirus (Zimmermann et al., 2011; Sakudo et al., 2016; Bunz et al., 2018), feline calicivirus (Aboubakr et al., 2018, 2020; Nayak et al., 2018; Yamashiro et al., 2018), murine norovirus (Bae et al., 2015; Lacombe et al., 2017; Park and Ha, 2018), and Tulane virus (Min et al., 2016; Lacombe et al., 2017). In these studies, the viruses were treated in various solutions or on various surfaces, including food, but none of these studies considered virus inactivation for water decontamination. Only two studies in the CAP-virus field have assessed the inactivation of viruses in larger volumes of water (Guo et al., 2018; Filipić et al., 2019), and only two have reported on the applications of CAP for inactivation of plant viruses, including potato virus Y (PVY; Filipić et al., 2019) and tomato mosaic virus (Hanbal et al., 2018).

Here we show that CAP can completely inactivate PMMoV in water samples after only 5 min, as all three independent treatments completely abolished infection of the test plants by the treated viruses. Complete inactivation of PMMoV was also achieved in two of three independent 3-min treatments, while in the third one, virus infectivity was partially decreased, with only two of seven plants infected. Such rapid and efficient inactivation is very encouraging considering the high resilience of PMMoV, which remains infective even after passing through the gastrointestinal tract or after various water treatments (Zhang et al., 2006; Kitajima et al., 2018; Bačnik et al., 2020; Tandukar et al., 2020). In addition, the concentrations of PMMoV used here (10^5^ cp μL^–1^) are much higher than those expected and reported in wastewater and other environmental water samples for PMMoV (Kitajima et al., 2018; Bačnik et al., 2020). Thus, it is likely that the times needed to inactivate PMMoV at environmentally relevant concentrations would be even shorter. Also, the water samples treated in this study contained a small amount of organic material that originated from the plant debris in the homogenates used to spike the tap water. Although this level of organic “pollution” is low, it might have protected the viruses from the CAP, which would mean that less polluted samples would require even shorter treatment times for successful virus inactivation, as confirmed in our previous study in which PVY was treated with the same CAP source (Filipić et al., 2019). PVY in water samples containing organic plant material (i.e., more organically polluted samples) was inactivated after only 5 min, while even shorter time of only 1 min was needed for its inactivation in tap water without any organic residues (i.e., less organically polluted samples). Despite morphological differences between PMMoV and PVY (e.g., rigid rod vs. flexible filament), these data show that very short treatment times that are in the range of only a few minutes (i.e., ≤5 min) are needed for inactivation of both of these virus types, which indicates that CAP can be the treatment of choice even for other viruses in water matrices. This is also supported by a study in which different CAP source successfully inactivated morphologically diverse viruses in water samples (Guo et al., 2018), which included bacteriophages MS2 and φ174 (icosahedral morphology) and bacteriophage T4 (very complex morphology: head with hemi-icosahedral ends, cocylindrical contractile tail, six fibers; Leiman et al., 2003). Other groups have shown the inactivation potential of CAP for additional viruses; however, they all either treated different non-aqueous liquids (e.g., buffers, mediums) containing the viruses, or they worked on inactivation of smaller volumes of up to few hundred microliters, and usually both (reviewed by Filipić et al., 2020).

To confirm that the inactivation of PMMoV here was a result of the CAP treatment, several control treatments were included, such as stirring, exposure of samples to gas only, and to H_2_O_2_. No viral inactivation was seen for H_2_O_2_ treatments at concentrations equivalent to those measured during the CAP treatments, as well as other control treatments. This means that the individual components of the treatment (i.e., H_2_O_2_, gas, stirring) did not inactivate PMMoV, and thus the complex environment generated as a result of the CAP treatment was responsible for PMMoV inactivation. In general, CAP produces high amounts of RONS, such as H_2_O_2_, where their exact composition depends on the experimental conditions. Each RONS has a different affinity toward a given molecule. For instance, singlet oxygen, which was shown to be the main inactivation agent for feline calicivirus (Aboubakr et al., 2016; Yamashiro et al., 2018) and bacteriophage T4 (Guo et al., 2018), reacts with tyrosine, tryptophan, cysteine, and guanine, and causes oxidative modifications of histidine residues and a shift in the molecular mass of methionine residues (Aboubakr et al., 2018; Guo et al., 2018). As well as singlet oxygen, ozone, H_2_O_2_, ONOOH, ONOO^–^, and NOx also have antiviral effects, which depend again on the unique experimental design (see review by Filipić et al., 2020). To date, all researchers in the CAP-virus field agree that RONS are the most important CAP factors responsible for virus inactivation, whereby other properties, such as UV radiation, appear to only have minor effects or no effects on the targeted viruses.

Reactive oxygen and nitrogen species are useful when dealing with inactivation of pathogenic organisms. However, on the other hand, it is known that RONS can induce oxidative damage to cells, which can lead to mutations, and thus the etiology of a wide variety of human diseases, such as chronic-inflammation-related disorders, carcinogenesis, neurodegeneration, and aging (Sharma et al., 2016). Therefore, it is very important to define the safety of CAP-treated water, especially if the water is intended to be reused for irrigation, drinking, or similar. The present study shows that none of the applied CAP treatment conditions induced cytotoxic or genotoxic effects in the HepG2 cell line. These findings of the *in-vitro* cytotoxicity and genotoxicity assessments indicate that no harmful by-products were formed in excess in these samples treated with CAP under these study conditions.

In addition to evaluation of the CAP influence on virus infectivity and the potential toxic effects of CAP-treated water, the present study was also aimed at the determination of the modes of viral inactivation. We observed changes in the capsid integrity by TEM after all of the CAP treatments, in contrast to the control treatments, which had no such effects. There were greater numbers of broken or damaged virus particles in the CAP-treated PMMoV samples, compared to the PMMoV-PC. Structures or aggregates were seen after 1-min treatments (Figure 2B), which were most likely indicative of early protein degradation; no such structures were seen for PMMoV-PC or the 5-min and 3-min CAP-treated PMMoV samples. In addition, the CAP treatment also had detrimental effects on the viral genomic RNA, as shown by RT-PCR (Figure 3). These data showed RNA degradation in all of the CAP-treated samples in at least two out of the three selected genomic regions amplified. In some cases of virus treatments using different CAP approaches, either protein or genetic material were damaged, while in others, both were affected (reviewed by Filipić et al., 2020). There have been only two studies in which CAP influenced both protein and nucleic acids, with the main mode of inactivation proposed to be protein damage followed by degradation of nucleic acids (Yasuda et al., 2010; Aboubakr et al., 2018). We observed that the CAP treatments can damage both virus protein and RNA, most likely by CAP-produced RONS, which are known to oxidize organic materials, including proteins and nucleic acids (Mittler, 2017). To determine the exact mechanisms of how CAP damages and targets PMMoV protein and RNA, further studies will be needed.

Clean water is one of the biggest challenges we are facing today. High water demand is generating an increased need for water reuse, which is a huge problem, as 80% of wastewater that often contains pathogenic viruses is not treated before it is released back into the environment (United Nations World Water Assessment Programme/Un-Water [WWAP/UN-Water], 2018). This is why there is an urgent need for environmentally friendly methods to successfully clean polluted waters. The present study is the first to investigate the CAP effects on PMMoV, and the emphasis of this study is water decontamination. Despite its high stability, the CAP treatment completely inactivated high concentrations of PMMoV in water samples after only 5 min of treatment, as measured by infectivity assays in test plants, without producing any cytotoxic or genotoxic by-products. Based on these data, we can conclude that CAP represents a very important water treatment tool that can also be used for inactivation of other pathogenic viruses, such as enteric viruses, which should ultimately lead to lower rates of human infections, and reduced crop losses. To further improve the decontamination potential of CAP, additional tests are needed, which include accurate CAP characterization and examination of its scale-up potential.

## Data Availability Statement

The raw data supporting the conclusions of this article will be made available by the authors, without undue reservation.

## Author Contributions

AF, DD, JŽ, IG, and MR conceived the study. AF performed the experiments and analyzed the data, with the exception of TEM, which was performed by MT, and assessment of cytotoxicity and genotoxicity, which was performed by AŠ and supervised by BŽ. GP and MM designed the CAP device. AF and IG wrote the manuscript and all of the authors revised it. All authors contributed to the article and have approved the submitted version.

## Conflict of Interest

The authors declare that the research was conducted in the absence of any commercial or financial relationships that could be construed as a potential conflict of interest.

## Footnotes

1 https://talk.ictvonline.org/ictv-reports/ictv_online_report/positive-sense-rna-viruses/w/virgaviridae/672/genus-tobamovirus
